# ‘Hajj: what it means for general practice’

**DOI:** 10.3399/bjgpopen18X101493

**Published:** 2018-04-18

**Authors:** Faraz Mughal, Carolyn A Chew-Graham, Ahmad Saad

**Affiliations:** 1 GP and Honorary Research Fellow, Academic Primary Care, University of Warwick, Coventry, UK; 2 NIHR In-Practice Fellow, Research Institute for Primary Care and Health Sciences, Keele University, Keele, UK; 3 Professor of General Practice Research, Research Institute for Primary Care and Health Sciences, Keele University, Keele, UK; 4 Director, Ihsan Institute for Arabic and Islamic Studies, London, UK

**Keywords:** Hajj, General Practice, Muslim

Annually, about 20 000 British Muslims journey to Saudi Arabia to undertake the Hajj, the greater Muslim pilgrimage.^1^ The National Travel Health Network and Centre advises intended pilgrims to see their GP 6 weeks prior to travel, to review their health needs and fitness to perform Hajj.^2^ With rising multimorbidity, polypharmacy, and risk of infectious disease, it is important for those in general practice to understand what Hajj involves, in order to provide culturally and religiously sensitive and appropriate medical advice and support.

## What is Hajj?

Hajj is the fifth pillar of Islam and is described in the Quran, as Almighty God says: 'Pilgrimage to this House is an obligation by God upon whoever is able among the people' (3:97).^3^ It is obligatory for every Muslim adult with mental capacity to perform the Hajj once in a lifetime, if reasonably able to do so without excessive hardship. The pilgrimage lasts 5 days, although pilgrims usually travel for longer. The Hajj occurs 10 days earlier each year (adhering to the lunar calendar), and in 2018 it is estimated to start around the 19 August.

The word 'Hajj' means to travel to the holy city of Mecca with the intention of performing certain rituals and visiting certain places at a specific time of the year. The actions of Hajj are specified and detailed in the books of jurisprudence. During Hajj days, millions of pilgrims move and worship within a small area of around 12 kilometers, in a display of dedication, universal brotherhood, and gratitude. An accepted Hajj brings reward no less than Paradise, as related from the Prophet Muhammad himself.^4^

The rites of Hajj are physically demanding, involving travelling in heat and among large crowds, in addition to engaging in demanding and draining physical rituals, which are lengthened by the masses of pilgrims, and occur in confined spaces (Hajj tents at Mina can hold up to 100 pilgrims each). Hajj is the largest annual gathering of people globally (1.86 million in 2016), and thus one can appreciate the challenge of performing individual rites among such a mass gathering.^5^

## The virtue of Hajj

Hajj is a deeply profound and spiritual act of worship that Muslims comprehensively prepare for, and is unique in that it involves the use of one’s body and wealth. Some spend years seeking the financial means to travel, and others fulfil rights of deceased relatives, through performing Hajj on their behalf. Pilgrims from all countries, whether a chief executive officer from the US or a farmer from Afghanistan, stand shoulder to shoulder in prayer, undertaking the Hajj rites, covered in sheets of cloth signifying equality and instilling humility in front of God.

## Health burden of Hajj

In 2017, Saudi Arabia’s Ministry of Health declared that there were 643 deaths occurring at Hajj. Of these deaths, 18% were attributed to heart failure, 15% to myocardial infarction, 3% sepsis, and 2% heatstroke.^6^ Pilgrims may present to health centres with a variety of illnesses; respiratory disease accounts for 61% of presentations, musculoskeletal problems 18%, dermatological 15%, and gastrointestinal 13%. One fifth of patients present with multiple problems.^7^

## Medical exemptions in performing Hajj

Physical health status is a condition considered as part of one’s ability to perform Hajj according to Shaykh Nur Al-Din ‘Itr, a scholar of international authority on Hajj.^8^ If a person has permanent disability, significant paralysis, or would experience difficulty travelling due to old age, an individual can be chosen to deputise to perform Hajj on their behalf.^8^ In temporary medical illness, a deputy cannot be selected; rather, one needs to wait until the medical condition improves to be deemed fit to perform Hajj by a clinician.

Clinicians should know that patients may still travel against medical advice, choosing to trust and rely on God. However, accurate medical advice must be given so that an informed decision can be made.

## Pre-Hajj advice

Box 1 highlights advice that should be given prior to the patient embarking on the Hajj journey.

**Box 1.** Advice to be given pre-Hajj

Preparation A progressive build-up of physical activity prior to the pilgrimage is advisable, to condition the body and mind for the physical and mental demands of the pilgrimage. Walking a few miles a day would be sufficient preparation to optimise exercise tolerance. Transport has improved considerably, with efficient train and bus routes now available along parts of the pilgrimage course for those who find walking long distances difficult.
Vaccinations^9^ Anyone travelling for Hajj should be adequately immunised. A certificate of the quadrivalent meningococcal ACWY vaccine is required for visa attainment. Those at high risk should be offered the influenza vaccine. Pneumococcal, typhoid, hepatitis A, hepatitis B, MMR, and polio immunisations should all be up to date.
Delaying menstruation Women may wish to delay their menses using medication.
Medication and list of drugs^10^ Pilgrims with chronic disease must carry sufficient medicines for the journey and prescription details. A diagnostic summary small enough to fit into a travel pouch would enable a rapid assessment in the 141 established primary medical centres or 24 hospitals within the immediate vicinity of the Hajj.
Diabetes Pilgrims with diabetes mellitus need to be advised to eat regular meals, check blood sugars often, and maintain medication compliance. If on insulin, they will need a letter for transport of needles and syringes through airports. They should be reminded about hypoglycaemic awareness, and should carry appropriate foods when performing the pilgrimage rites to prevent exertion-induced hypoglycaemia. Good footwear is important, however the type must be religiously permitted according to the pilgrim’s school of Islamic law. Blistering heat in the day is common while walking, and thus feet are prone to blisters. If bare-footed (not recommended) then the risk of infection, burns, and cuts, especially in pilgrims with diabetes, is high.
Heat The heat during Hajj (>40 degrees celsius in summer months) carries risks of heat exhaustion and heatstroke. To avoid this, patients should be reminded of simple practical measures: avoiding prolonged exposure to the sun, drinking and carrying plenty of fluid, using unscented sunscreen, and keeping one’s head covered where possible (men cannot directly cover their heads during Hajj, but can use a white umbrella to deflect sunlight).
Public health awareness During the Hajj, men may get their head shaved and women may shorten their hair. The razors used by street barbers may not always be clean and thus pilgrims should insist on a new razor blade, to reduce the chance of blood-borne virus exposure (for example, HIV, and hepatitis B and C).

## Post-Hajj considerations

The mass gathering of Hajj makes susceptibility to airborne disease likely. The spread of respiratory tract infections is common, and on return from Hajj, the primary care doctor should consider tuberculosis, atypical pneumonia, and Middle East respiratory syndrome coronavirus in patients with flu-like symptoms.^2,7^

Primary care clinicians need also to be vigilant for symptoms or signs of hepatitis, malaria, meningitis, and hydatid disease in patients presenting with acute illness on returning from Hajj.^9^

## Implications for policy

Clinicians can signpost patients to detailed online information for Hajj, but there are no readily accessible patient leaflets that summarise comprehensive health advice for those who intend to travel for Hajj.^1,2^ These could be administered in primary care consultations and should be available in different languages. The present authors feel this should be a priority for NHS England, Public Health England, and the Muslim Council of Britain.

## Implications for practice

Hajj is the largest annual mass gathering event internationally.^5^ It is therefore important for primary care practitioners to be familiar with how to appropriately advise patients intending to travel for the pilgrimage. Patients with complex health needs and polypharmacy must be counselled adequately on their fitness to travel for the Hajj.
